# Science and Self: Aging as Woven

**DOI:** 10.1093/geroni/igaa057.2327

**Published:** 2020-12-16

**Authors:** Helen Kivnick

**Affiliations:** University of Minnesota, St. Paul, Minnesota, United States

## Abstract

Gerontology is a field both scientific and practice-based. Aging, the subject of this field, is an experience in which all human beings participate. But scientific pillars of objectivity, quantifiability, control, and external validity have long mitigated against gerontological scholars effectively moving back and forth between professional scholarship and practice, on the one hand, and personal experience, on the other. Qualitative research approaches, informed by the humanities and arts, utilize alternative ways of knowing that, when added to positivistic science, enable us to construct a body of gerontological knowledge that is robust and useful, and that also incorporates wisdom. Aging, wisdom, and integrality—these all matter. Although often mischaracterized, Erikson’s theory of healthy psychosocial development throughout the life-cycle (Erikson, Erikson, & Kivnick, 1986) weaves these constructs together in ways that can meaningfully inform professional and personal experiences of gerontology. This presentation illustrates one aging gerontologist’s engagement with such weaving.

